# Smoking habits and incidence of cardiovascular diseases in men and women: findings of a 12 year follow up among an urban Eastern-Mediterranean population

**DOI:** 10.1186/s12889-019-7390-0

**Published:** 2019-08-05

**Authors:** Parisa Amiri, Kamyar Mohammadzadeh-Naziri, Behnood Abbasi, Leila Cheraghi, Sara Jalali-Farahani, Amir Abbas Momenan, Atieh Amouzegar, Farzad Hadaegh, Fereidoun Azizi

**Affiliations:** 1 Research Center for Social Determinants of Health, Research Institute for Endocrine Sciences, Shahid Beheshti University of Medical Sciences, Tehran, Iran; 2 Department of Nutrition, Science and Research Branch, Islamic Azad University, Tehran, Iran; 3 Biostatistics Department, Research Institute for Endocrine Sciences, Shahid Beheshti University of Medical Sciences, Tehran, Iran; 4 Students’ Research Committee, Shahid Beheshti University of Medical Sciences, Tehran, Iran; 5 Prevention of Metabolic Disorders Research Center, Research Institute for Endocrine Sciences, Shahid Beheshti University of Medical Sciences, Tehran, Iran; 6 Endocrine Research Center, Research Institute for Endocrine Sciences, Shahid Beheshti University of Medical Sciences, P.O.Box: 19395-4763, Tehran, Iran

**Keywords:** Smoking habits, Cardio-vascular outcomes, Iran, TLGS

## Abstract

**Background:**

Despite the strong association of smoking with cardiovascular disease (CVD) and cerebral stroke, the consequences of smoking have not been elucidated among Iranian populations. This study aimed to assess sex-specific incidence of CVDs among an urban Iranian population with different smoking habits.

**Methods:**

Participants were recruited from the Tehran Lipid and Glucose Study (TLGS). Data on socio-demographic features and smoking habits from a sample of 10,400 individuals (4378 men and 6022 women), aged ≥20 years without prior CVD history were analyzed. Participants were followed up for 12 years for incidence of CVD/CHD events. Men were categorized in six groups, including never-, passive, ex-, passive and ex-, occasional and daily smokers. Women were categorized in three groups, i.e. never smokers, passive smokers and ever smokers. Using cox regression model, adjusted hazard ratios (HRs) of incident CVD/CHD were calculated for each group, given never smokers as the reference.

**Results:**

In men, HR of CVD was 1.13 (95%CI: 0.80–1.59) in passive smokers, 1.23 (95%CI: 0.91–1.66) in ex-smokers, 1.46 (95%CI: 0.90–2.36) in passive and ex-smokers, 2.33 (95%CI: 1.25–4.33) in occasional smokers and 2.05 (95%CI: 1.57–2.67) in daily smokers. In smokers of ≥21 cigarettes/day, HR of CVD was 3.79 (95%CI: 2.25–6.37), with less risk observed in those who smoked lesser numbers of cigarettes/day. Quitters of ≥15 years were almost risk free. In women, none of the HRs of CVD/CHD were significant.

**Conclusion:**

An increased risk of incidence of CVD/CHD was found in current male smokers. To confirm and further elaborate these findings, more data of sex-specific studies are required from culturally diverse urban and rural areas of Iran.

## Background

Cardiovascular diseases (CVDs) are among the most prevalent non-communicable diseases (NCDs) responsible for 31% of all global mortality [1]. Due to fast increasing changes in human lifestyles, prevalence of CVDs is markedly increasing in both the developed and developing countries [2]. According to the recent assessment of GBD (Global Burden of Disease), an estimated 422.7 million individuals suffer from CVDs and 17.9 million annual deaths are attributed to these diseases [3]. The burden of CVDs and its related risk factors as well as its rising trend in Eastern Mediterranean communities, including Iran is very alarming [2]. Ischemic heart disease was found to be the main cause of death in Iran [4], with a 5.9% prevalence for coronary heart disease (CHD) and 3% for stroke, reported recently by a prospective study [5]. Regarding the high prevalence and incidence of CVDs, there has been an increasing trend to ascertain the main determinants and underlying causes of these diseases. Meanwhile, the most prominent risk factors of CVDs are obesity, low physical activity, glucose intolerance, hypertension, emotional stress and smoking [6].

Smoking is the second major modifiable risk factor of CVDs [7] which directly harms and affects cardiac vasculature, and also contributes to development of other cardiovascular risk factors, such as glucose intolerance, dyslipidemia and thrombus formation [8]. Iran has been the most successful country in implementing world health organization (WHO) measures for tobacco control in the Eastern Mediterranean region [9]; according to the latest reports, however, smoking prevalence is high and about a fifth of Iranian men are still smokers [4]. Aside from active smoking, passive smoking or exposure to environmental tobacco smoke (ETS) directly plays a substantial role in CVDs as well [10], and can aggravate the contributing risk factors, viz. metabolic syndrome, vascular inflammation, thrombus formation and atherosclerosis [11].

Despite the relationship between smoking and CVDs being well documented [12], because of geographical variations in patterns, the burden of cardiovascular risk factors [13–15] and the lack of longitudinal studies among Iranians, especially in women and passive smokers [16–18], data available cannot be generalized to all Iranians. The Tehran Lipid and Glucose Study (TLGS), is a large prospective cohort aimed to investigate the risk factors of NCDs among Iranian populations. However, previous studies on the association of smoking with CVDs among the TLGS populations did not include passive and female smokers [19–21]. Thus, the current study, for the first time in Iran, aims to assess the association between smoking and the incidence of CVD/CHD from two aspects, i.e. the smoking habits and smoking intensity among Tehranian men and women.

## Methods

### Study population

The TLGS is an ongoing community-based study initiated in 1999, and designed to be continued for at least 20 years. The main goal of study is to assess risk factors and different aspects of life style in relation with NCDs among a representative urban Tehranian population [22]. Details of its goals and design have been published previously [22, 23].

**Fig. 1:**
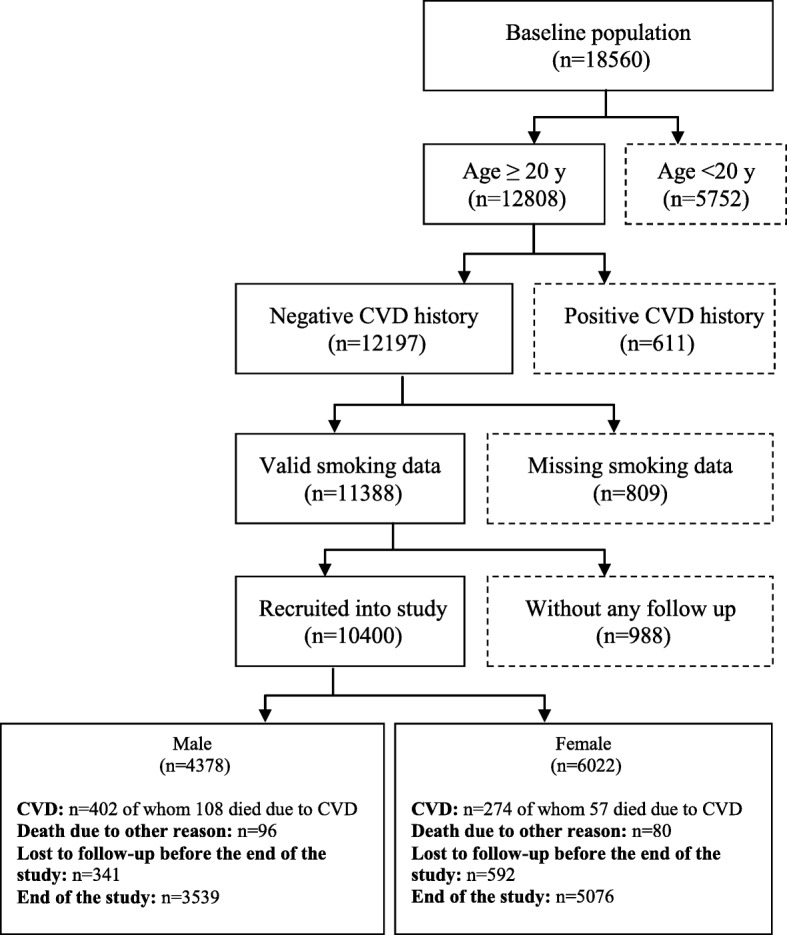
Flow diagram of participants recruitment. Dashed boxes represent exclusion. CVD. Cardiovascular diseases

The current analysis has been conducted on all TLGS participants who participated in the study between 1999 and 2002; those aged under 20 years, those with positive CVD history, those with missing smoking data and those lost to follow up were excluded. A final sample of 10,400 adult individuals (4378 men and 6022 women) was followed for a median of 12 years. The sampling frame of the current analysis is shown in Fig. 1. The study was approved by the research ethics committee of the Research Institute for Endocrine Sciences (RIES), Shahid Beheshti University of Medical Sciences. Written informed consents were obtained from all participants.

### Baseline measurements

Data on socio-demographic information (age, education level, marital status and occupation), smoking habits and exposure to ETS and any familial history of NCDs were collected by trained interviewers. For physical activity, data was collected using a validated, reliable questionnaire entitled the Modifiable Activity Questionnaire (MAQ) [24]. Psychometric properties of the Iranian version of MAQ have been assessed in the TLGS population and showed acceptable validity and high reliability [25]. Weight was measured by digital scales and was recorded to the nearest 100 g, without shoes and with light clothing. Height was measured standing, without shoes. Body mass index (BMI) was calculated by dividing weight (kg) by square of height (m^2^). After participants rested for 15 min, a physician measured blood pressure using a mercury sphygmomanometer with appropriate cuff size for each individual. Systolic and diastolic blood pressure were measured twice, at least 30-s apart and the mean of the two measurements was recorded as the participant’s blood pressure. After 12 to 14 h of overnight fasting, blood samples were collected between 7:00 and 9:00 AM to be sent to TLGS research laboratory and biochemically analyzed on the same day. Levels of fasting blood glucose (FBS), total cholesterol, high density lipoprotein (HDL-c) and triglycerides (TG) were enzymatically measured. Low density lipoprotein (LDL-c) was calculated using Friedewald formula [26]. The detailed process of socio-demographic, anthropometric and biochemical assessments has previously been published elsewhere [22, 23].

### Definitions of smoking groups

Male participants were categorized into the six following groups: 1) never smokers, 2) passive smokers (exposure to ETS), 3) ex-smokers (quitter since at least a month before interview), 4) passive and ex-smokers (quitters exposed to ETS), 5) active occasional smokers (non-routine smokers) and 6) active daily smokers. In another grouping pattern, based on number of cigarettes smoked per day, active male smokers (whether occasional or daily) have been further subdivided in to three groups: ≤10, 11–20 and ≥ 21 cigarettes per day. Ex-smokers were also subcategorized into those who quit smoking in less than 15 years, and those that had quit 15 years ago or earlier, regardless of passive smoking. Female participants were categorized as never smokers, passive smokers (same as males) and ever smokers (active and ex-smokers pooled together). Different categorization in men and women in the current study was mainly due to the limited number of women who reported themselves as active (*n* = 146) or past smokers (*n* = 94) and consequently the limited number of CVD events that occurred in these groups (*n* = 15) through the study period.

### Outcome measures

Participants were followed up annually for any cardiovascular event through phone call interviews by trained nurses. A physician confirmed the diagnosis by home visits or data collection from the medical file or death certification, in case of mortality. A committee consisting of an internist, endocrinologist, cardiologist, epidemiologist and other professionals when needed, confirmed the outcome data; CHD event was defined as any definite myocardial infarction (MI) (diagnostic electrocardiogram (ECG) and biomarkers), probable MI (positive ECG findings and cardiac symptoms plus missing biomarkers or positive ECG findings plus equivocal biomarkers), sudden cardiac death, unstable angina pectoris (new cardiac symptoms or changing symptom pattern and positive ECG findings with normal biomarkers) and angiographically proved coronary artery disease [27]. The definition of CVD event was that of CHD, plus fatal or non-fatal stroke (defined as a new neurological deficit lasting ≥24 h). Details of outcome data analysis and definitions have been described elsewhere [22, 23, 28].

### Statistical analysis

All analyses and baseline characteristics are classified by sex. Continuous variables are expressed as mean ± (SD) for normally distributed and as median (Q1-Q3) for skewed ones. To compare continuous variables between different CVD groups, Student t-test and Mann-Whitney test were used for normally distributed and skewed variables, respectively. In different smoking groups, continuous variables were compared by ANOVA in normally distributed- and by Kruskal-Wallis test in skewed ones. Categorical variables are expressed as number (%) and have been compared with Chi-square test, both within different smoking groups and CVD groups. Cox proportional hazard model was used to study the association between different smoking patterns and cardiovascular outcomes. The follow-up duration was defined as the period starting from entrance to study and ending in occurrence of any predefined CVD/CHD event or any attributed mortality or censoring, whichever happened first. Censoring was defined as loss to follow-up or mortality, not attributable to CVD or CHD. Univariate regression model was first performed for all potential confounding factors and those with *p*-values less than 0.2 were selected for multivariate regression. Adjustment of potential confounding factors was conducted in three cumulative models; the first model was adjusted for age, the second for age and cardio-metabolic risk factors (BMI, systolic and diastolic blood pressure, fasting blood sugar, total cholesterol and triglycerides) and the third model was adjusted for all the above mentioned variables, plus socio-economic factors (education level, reference: college degree; marital status, reference: married and occupation, reference: employed). Within each model, adjusted hazard ratios (HRs) of outcome events were estimated in men and women with 95% confidence intervals (95% CIs) for each smoking pattern, given never smokers as reference. Proportionality assumption of Cox models was assessed using the Schoenfeld residual test and confirmed. Statistical analyses were performed by STATA software version 12, and SPSS software version 15. *P*-values below 0.05 were considered statistically significant.

## Results

A total of 10,400 CVD free participants, aged ≥20 years with complete data on smoking status, were followed during 12 years for occurrence of CVD or CHD events. Mean ages of participants at baseline were 42.73 ± 15.30 and 40.33 ± 14.00 years in men and women, respectively. Over a median 12 year follow up, incidence rate of CVD was 10.6 (95% CI: 9.6–11.7) and 5.1 (95% CI: 4.5–5.8) per 1000 person years, in men and women respectively. Incidence rate of CHD was 9.0 (95% CI: 8.1–10.0) in men and 4.4 (95% CI: 3.9–5.0) in women, per 1000 person years.

Table 1 shows baseline characteristics and CVD/CHD incidence of study participants based on their smoking status. In men, occasional smokers had the highest levels of BMI, TG, total cholesterol and LDL-C; lowest levels of physical activity were found among daily smokers. In women, ever smokers had higher levels of illiteracy compared to never- and passive smokers. The highest rate of CVD/CHD incidence was among ex-smokers in males and among ever smokers in female participants.

**Table 1:** Baseline characteristics and CVD/CHD incidence of study participants based on smoking status

	Men (*n* = 4378)						*P* value	Women (*n* = 6022)			*P* value
	Never smoker (*n* = 1895) (43.3%)	Passive smoker (*n* = 831) (19%)	Ex-smoker (*n* = 454) (10.4%)	Passive & ex-smoker (*n* = 207) (4.7%)	Occasional smoker (*n* = 98) (2.2%)	Daily smoker (*n* = 893) (20.4)		Never smoker (*n* = 4531) (75.2%)	Passive smoker (*n* = 1251) (20.8%)	Ever smoker (*n* = 240) (4%)	
Age	43.97 ± 16.4	36.64 ± 13.1	51.13 ± 15.7	41.40 ± 13.1	39.56 ± 12.9	42.17 ± 12.6	< 0.001	40.74 ± 14.2	37.72 ± 12.9	46.28 ± 13.3	< 0.001
Education level n (%)											
Illiterate	107(5.6)	17(2.1)	37(8.1)	10(4.8)	3(3.1)	28(3.1)	< 0.001	490(10.8)	111(8.9)	46(19.2)	< 0.001
Lower than diploma	663(35.0)	230(27.7)	210(46.3)	72(34.8)	34(34.7)	364(40.9)		1828(40.4)	517(41.4)	88(36.7)	
Diploma	731(38.6)	426(51.4)	129(28.4)	95(45.9)	40(40.8)	383(43.0)		1695(37.4)	502(40.2)	84(35.0)	
Higher	394(20.8)	156(18.8)	78(17.2)	30(14.5)	21(21.4)	116(13.0)		515(11.4)	120(9.6)	22(9.2)	
Marital status n (%)											
Single	380(20.1)	259(31.2)	40(8.8)	28(13.5)	18(18.4)	131(14.7)	< 0.001	573(12.6)	204(16.3)	12(5.0)	< 0.001
Widowed/divorced	14(0.7)	3(0.4)	8(1.8)	1(0.5)	1(1.0)	9(1.0)		428(9.4)	74(5.9)	48(20.0)	
Married	1501(79.2)	569(68.4)	406(89.4)	178(86.0)	79(80.6)	753(84.3)		3530(78.0)	973(77.8)	180(75.0)	
Occupation n(%)											
Unemployed	231(12.2)	98(11.8)	37(8.2)	12(5.8)	12(12.2)	55(6.2)	< 0.001	2832(84.6)	1049(83.9)	197(82.1)	< 0.001
Unemployed with income	374(19.7)	35(4.2)	137(30.2)	23(11.1)	10(10.2)	102(11.4)		192(4.2)	28(2.2)	19(7.9)	
Employed	1289(68.1)	698(84.0)	279(61.6)	172(83.1)	76(77.6)	735(82.4)		507(11.2)	174(13.9)	24(10.0)	
Physical activity n(%)											
Low	1036(56.9)	446(55.7)	264(59.6)	101(51.6)	54(55.7)	606(68.2)	< 0.001	2533(59.4)	710(59.3)	143(60.8)	0.655
Moderate	307(16.9)	145(18.1)	59(13.3)	34(17.3)	22(22.7)	116(13.0)		619(14.5)	167(14.0)	26(11.1)	
High	477(26.2)	210(26.2)	120(27.1)	61(31.1)	21(21.6)	167(18.8)		1115(26.1)	320(26.7)	66(28.1)	
BMI (kg/m^2^)	25.95 ± 4.0	25.71 ± 4.2	26.22 ± 4.0	26.28 ± 3.9	26.55 ± 4.5	25.12 ± 4.2	< 0.001	27.39 ± 5.1	27.35 ± 5.2	27.94 ± 4.3	0.147
FBS (mmol/l)	5.43 ± 1.60	5.19 ± 1.21	5.70 ± 1.90	5.46 ± 1.74	5.45 ± 1.64	5.32 ± 1.63	< 0.001	5.36 ± 1.80	5.28 ± 1.70	5.53 ± 2.12	0.094
TG (mmol/l)*	1.67 (1.11–2.42)	1.63 (1.10–2.41)	1.83 (1.31–2.60)	1.65 (1.15–2.56	1.93 (1.34–2.70	1.80 (1.20–2.61)	< 0.001	1.47 (0.99–2.21)	1.40 (0.96–2.07)	1.55 (1.08–2.23)	0.004
Chol (mmol/l)	5.17 ± 1.08	5.04 ± 1.12	5.35 ± 1.14	5.11 ± 1.31	5.44 ± 1.06	5.25 ± 1.14	< 0.001	5.39 ± 1.26	5.23 ± 1.19	5.55 ± 1.26	< 0.001
HDL-C (mmol/l)	1.01 ± 0.24	0.98 ± 0.23	0.98 ± 0.22	0.99 ± 0.23	0.97 ± 0.26	0.96 ± 0.25	< 0.001	1.17 ± 0.29	1.16 ± 0.30	1.13 ± 0.27	0.179
LDL-C (mmol/l)	3.29 ± 0.90	3.21 ± 0.95	3.45 ± 0.94	3.21 ± 0.97	3.52 ± 0.90	3.36 ± 0.96	< 0.001	3.43 ± 1.04	3.30 ± 0.96	3.62 ± 1.04	< 0.001
SBP (mmHg)	122.1 ± 18.9	118.04 ± 14.8	124.4 ± 19.7	119.5 ± 17.5	118.2 ± 16.7	114.6 ± 15.8	< 0.001	117.4 ± 19.4	115.3 ± 18.6	117.7 ± 20.4	0.002
DBP (mmHg)	78.6 ± 11.1	77.8 ± 10.2	78.1 ± 11.2	78.4 ± 11.6	78.1 ± 11.6	74.0 ± 10.46	< 0.001	76.8 ± 10.9	76.1 ± 10.4	76.2 ± 11.1	0.153
With CVD	161 (8.5)	42 (5.5)	61 (13.4)	19 (9.2)	11 (11.2)	104 (11.2)	< 0.001	213 (4.7)	46 (3.7)	15 (6.3)	0.133
With CHD	133 (7.0)	42 (5.1)	49 (10.8)	19 (9.2)	9 (9.2)	92 (10.3)	< 0.001	183 (4.0)	40 (3.2)	13 (5.4)	0.189

Normally distributed continuous variables are presented as mean ± SD and *p* value was calculated with ANOVA; * Skewed continuous variables are presented as median (Q1-Q3) and *p* value was calculated with Kruskal-Wallis; Categorical variables are presented as number (%) and *p* value was calculated with Chi-square test. *BMI* body mass index, *FBS* fasting blood sugar, *TG* triglycerides, *Chol* cholesterol, *HDL-C* high density lipoprotein-cholesterol, *LDL-C* low density lipoprotein-cholesterol, *SBP* systolic blood pressure, *DBP* diastolic blood pressure, *CVD* cardiovascular disease, *CHD* coronary heart disease. *P* < 0.05 has been considered significant

**Fig. 2:**
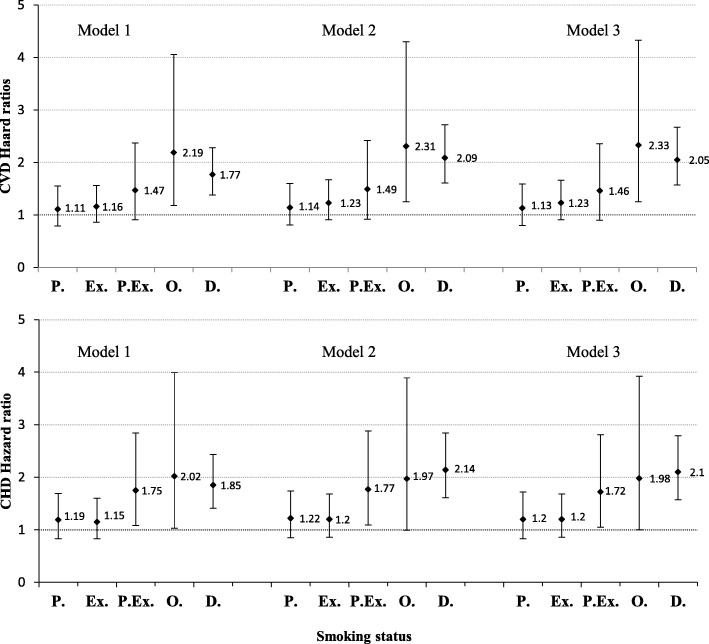
Hazard ratios and 95% confidence intervals (CI) for CVD/CHD incidence in men, among different smoking groups (Ref: never smoker). Tehran Lipid and Glucose study 1999–2010. Model 1. Adjusted for age. Model 2. Adjusted for age and cardio-metabolic risk factors including: body mass index, systolic blood pressure, diastolic blood pressure, triglyceride levels, cholesterol level and fasting blood sugar. Model 3. Adjusted for age, cardio-metabolic risk factors (as above) and socio-economic features including: education (Ref: college degree), marital status (Ref: married) and occupation (Ref: employed). P. Passive smoker; Ex. Ex-smoker; P.Ex. Passive and Ex-smoker; O. Occasional smoker; D. Daily smoker

Hazard ratios of CVD/CHD incidence among different smoking groups of men, are shown in Fig. 2. In men, compared with never smokers, even after adjusting for potential confounders (model 3), HRs of CVD incidence were 1.13 (95% CI: 0.80–1.59) in passive smokers, 1.23 (95% CI: 0.91–1.66) in ex-smokers, 1.46 (95% CI: 0.90–2.36) in passive and ex-smokers, 2.33 (95% CI: 1.25–4.33) in occasional smokers and 2.05 (95% CI: 1.57–2.67) in daily smokers. Adjusted HRs of CHD incidence were 1.20 (95% CI: 0.83–1.72) in passive smokers, 1.20 (95% CI: 0.86–1.68) in past smokers, 1.72 (95% CI: 1.05–2.81) in past and passive smokers, 1.98 (95% CI: 1.00–3.92) in occasional smokers and 2.10 (95% CI: 1.57–2.79) in daily smokers, all compared with never smokers.

**Fig. 3:**
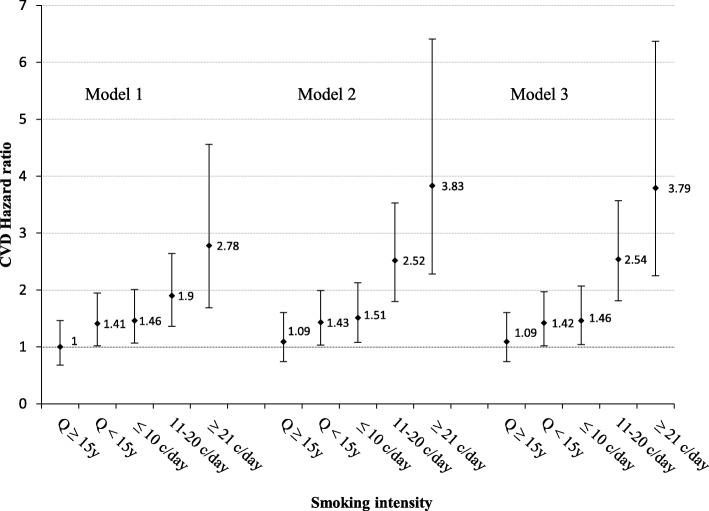
Hazard ratios and 95% confidence intervals (CI) for CVD incidence in male smokers and ex-smokers, based on the number of cigarettes smoked per day, or the time spent since the last quit, respectively (ref: never smoker). Model 1. Adjusted for age. Model 2. Adjusted for age and cardio-metabolic risk factors including: body mass index, systolic blood pressure, diastolic blood pressure, triglycerides level, cholesterol level and fasting blood sugar. Model 3. Adjusted for age, cardio-metabolic risk factors (as above) and socio-economic features including: education (Ref: college degree), marital status (Ref: married) and occupation (Ref: employed). Q ≥ 15y, Quit 15 years ago or before; Q < 15y, Quit in the last 15 years; ≤ 10 c/day, Smoking ≤10 cigarettes/day; 11–20 c/day, Smoking 11–20 cigarettes/day; ≥ 21 c/day, Smoking ≥21 cigarettes/day

Figure 3 illustrates HRs of CVD based on the number of cigarettes smoked per day or the time passed since last cessation, in active and ex-smoker males respectively. Adjusted for all potential confounders in the third model, HRs of CVD incidence were 1.09 (95% CI: 0.74–1.60) in quitters of ≥15 years ago, 1.42 (95% CI: 1.02–1.97) in quitters of < 15 years ago, 1.46 (95% CI: 1.04–2.07) in smokers of ≤10 cigarettes/day, 2.54 (95% CI: 1.81–3.57) in smokers of 11–20 cigarettes/day and 3.79 (95% CI: 2.25–6.37) in smokers of ≥21 cigarettes/day all compared with never smokers. As shown in Fig. 3, higher CVD risk was observed with higher numbers of cigarettes smoked per day. Male ex-smokers who had quit 15 years ago or earlier had the same CVD risk as never smokers.

**Fig. 4:**
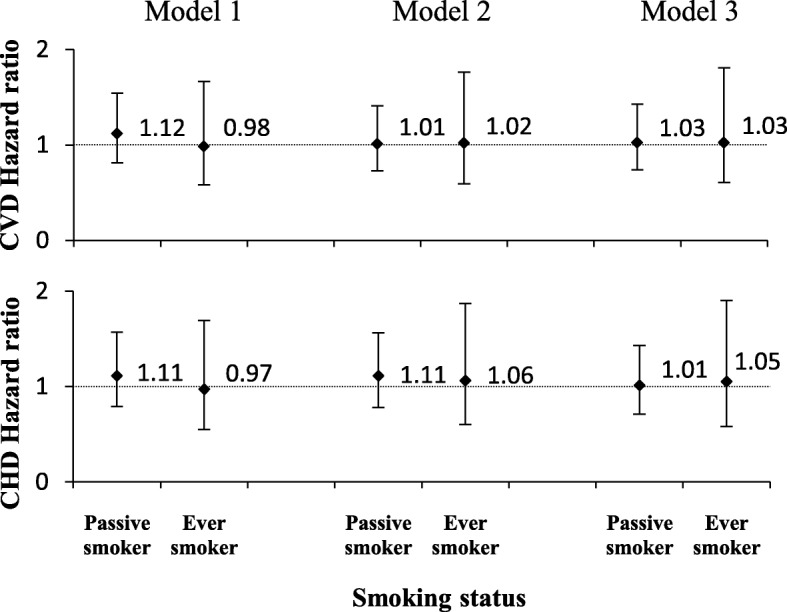
Hazard ratios and 95% confidence intervals (CI) for CVD/CHD incidence in women, among different smoking groups (Ref: never smoker). Tehran Lipid and Glucose study 1999–2010. Model 1. Adjusted for age. Model 2. Adjusted for age and cardio-metabolic risk factors including: body mass index, systolic blood pressure, diastolic blood pressure, triglycerides level, cholesterol level and fasting blood sugar. Model 3. Adjusted for age, cardio-metabolic risk factors (as above) and socio-economic features including: education (Ref: college degree), marital status (Ref: married) and occupation (Ref: employed)

Figure 4 shows hazard ratio of CVD/CHD among different smoking groups of women, in whom, HRs of CVD incidence were 1.03 (95% CI: 0.74–1.43) in passive smokers and 1.03 (95% CI: 0.61–1.81) in ever smokers. Hazard ratios of CHD incidence were 1.01 (95% CI: 0.71–1.43) in passive smokers and 1.05 (95% CI: 0.58–1.9) in ever smokers, compared with never smokers.

## Discussion

Our results showed that different smoking habits and higher rates of cigarette smoking increased the risk of CVD/CHD in men. In women, however, the relation between different smoking patterns and the risk of CVD/CHD was not established with these data. According to our findings, smoking rates were significantly higher among men than women, which is consistent with previous findings from Iran and other Middle Eastern countries. In this regard, a recent meta-analysis indicated that the prevalence of smoking in Iran ranged between 19.8 and 21.7% in men, while it was estimated to be 0.94 to 3.6% in women [18]. According to the latest WHO report (2017), the same pattern was also seen in other Middle Eastern countries including Kuwait (35.4% in men and 2.0% in women), Oman (12.3% in men and 0.1% in women) and Qatar (18.2% in men and 0.1% in women) [29]; gender differences however, were lower in East-Asian communities, including Japan (28.0% in men and 8.8% in women) [30] and Korea (37.6% in men and 4.9% in women) [31]; these gender differences in smoking were even less in Western countries such as Britain (19.1% in men and 15.7% in women) [32] and United States (15.8% in men and 12.4% in women) [33]. The geographical variation of smoking prevalence in men and women may be due to under-reporting of smoking in women of the Middle Eastern countries, where smoking is usually associated with social stigma [34].

Our findings regarding the prognostic value of current smoking both occasionally and daily in the prediction of CVD/CHD incidence are in agreement with the findings of Birgitte et al. who reported an age-adjusted relationship of active- and passive smoking with MI incidence in males and females [35]. Shields and Wilkins also reported that active daily smokers had a 60% higher risk of CHD incidence in Canada [36]. Several other studies also show that passive smoking may result in increment in the risk of MI [37–39]. Further analysis showed that the relation between smoking and risk of CVD followed a dose-dependent association, i.e. the “more cigarettes smoked daily, the higher the risk of CVD”, a finding in line with previous studies reporting that smoking intensity is significantly related with an increased risk of CVD [40–42]. Furthermore, the risk of CVD in men who had quit smoking for over 15 years, tends to be similar to that in never smokers, results in accordance with previous findings of the Surgeon General’s Report on the health benefits of smoking cessation, reporting that reduced risk of heart diseases may not show itself until ≥15 years after complete abstinence from smoking [43]. Shields and Wilkins also reported that at least 20 years of smoking cessation was required for the risk of heart diseases to normalize the risk of heart disease in former smokers [36].

Based on our findings, no association between various smoking habits and risk of CVD/CHD was found in females, findings in contrast with those of previous studies, reporting that smoking is the leading contributing factor in development and progression of CVDs in both men and women [44]. Furthermore, it is well established that smoking is associated with a wide range of risk factors which increase the risk of CVD, e.g. endothelial dysfunction, decreased insulin sensitivity, increased heart rate and blood pressure, hypercoagulable and hyper inflammatory status and altered serum lipoproteins and lipid related enzymes [45]. Some studies even propose that women may be more susceptible than men to some of the smoking-related CVD consequences and have a significant 25% increased risk for CHD, compared to their male counterparts [46–48]. The current findings regarding lack of association between smoking pattern and CVD/CHD outcomes in women may be due to the under reporting of their smoking habits. While smoking in men is considered a common habit, smoking in women is often seen as inappropriate and is associated with social stigma in developing countries [49]; these socio-cultural factors may raise questions about the accuracy and validity of self-reported data of smoking in women [34]. Another reason that may increase the risk of misreporting is the presence of other family members who usually accompany younger or female participant during the interview [50]. In this situation, some researchers found that women tend to conceal their smoking habits from family members, mainly because of the shame and stigma attached [51].

Regarding the strengths of the current study to the best of our knowledge, this is the first longitudinal study to assess the association of smoking habits with the risk of cardiovascular diseases among Iranian populations among women and passive smokers. Among the limitations of the study was the possible information bias of data collection using a questionnaire-based method. In addition the lack of significant findings in women which can be explained in light of the potential under-reporting of smoking as well as the lack of statistical power due to low number of current smokers; limitations which need to be further investigated in future studies in Iran.

## Conclusion

In conclusion, although an increased risk of CVD/CHD incidence was found in current male smokers, this finding was not supported by our data in women. This result might be attributed to under-reporting of smoking in female participants due to the existing social stigma regarding smoking of women in Iran. Our findings indicated the importance of smoking cessation to prevent cardio-vascular outcomes in an Iranian urban population. To confirm and further elucidate these findings, more studies are required in both urban and rural areas of Iran.

## Data Availability

Data would be available on the request of corresponding author based on TLGS rules.

## Abbreviations

BMI: Body mass index
CHD: Coronary heart disease
CVD: Cardiovascular disease
ECG: Diagnostic electrocardiogram
ETS: Environmental tobacco smoke
FBS: Fasting blood glucose
GBD: Global Burden of Disease
HDL-c: High density lipoprotein
HRs: Hazard ratios
LDL-c: Low density lipoprotein
MAQ: Modifiable Activity Questionnaire
MI: Myocardial infarction
NCDs: Non-communicable diseases
RIES: Institute for Endocrine Sciences
TG: Triglycerides
TLGS: Tehran Lipid and Glucose Study
WHO: World health organization
